# Supplementary material for: Analysis of Microorganism Colonization, Biofilm Production, and Antibacterial Susceptibility in Recurrent Tonsillitis and Peritonsillar Abscess Patients
Source: Int J Mol Sci. 2022 Sep 7;23(18):10273. doi: 10.3390/ijms231810273 (PMC9499404; doi:10.3390/ijms231810273)
Supplement: Supplementary file 1 [file ijms-23-10273-s001.zip › ijms-1869327-supplementary.pdf]

International Journal of *Molecular Sciences*

MDPI

Supplementary Tables

Table S1. Microorganisms isolated from the tonsillar crypts of 99 patients with RT.

Combinations of Isolated Strains	Count (n=99)
Normal oral microbiota	39
S. aureus	14
S. aureus + normal oral microbiota	7
S. aureus + *Candida* spp.	1
S. aureus + *Lactobacillus paracasei*	1
S. aureus + *Streptococcus parasanguinis*	1
S. aureus + *Staphilococcus epidermidis*	1
S. aureus + *Haemophilus influenzae*	1
S. aureus + *Klebsiella pneumoniae*	2
S. aureus + *Klebsiella oxytoca*	1
S. aureus + *Streptococcus agalactiae* + normal oral microbiota	1
S. aureus + *Klebsiella pneumoniae* + *Candida* spp. + normal oral microbiota	1
S. aureus + *Streptococcus pneumoniae* + normal oral microbiota	1
S. aureus + *Prevotella intermedius* + *Streptococcus oralis* + normal oral microbiota	1
S. aureus + *Streptococcus anginosus* + *Neisseria subflava* + *Haemophilus influenzae* + *Prevotella intermedius* + normal oral microbiota	1
Staphylococcus pseudintermedius	1
Staphylococcus capitis + *Aggregatibacter aphrophilus*	1
Streptococcus parasanguinis	1
Streptococcus anginosus	1
Streptococcus anginosus + normal oral microbiota	1
Streptococcus anginosus + *Candida* spp.	1
Streptococcus pyogenes + *Streptococcus pneumoniae*	1
Streptococcus pyogenes + *Morganella morganii*	1
Streptococcus oralis + normal oral microbiota	1
Streptococcus oralis + *Streptococcus mitis* + normal oral microbiota	1
Escherichia coli	2
Escherichia coli + *Staphilococcus epidermidis*	1
Klebsiella pneumoniae	5
Klebsiella pneumoniae + *Streptococcus pyogenes*	1
Klebsiella pneumoniae + *Candida* spp.	1
Klebsiella pneumoniae + *Streptococcus pyogenes* + *Candida* spp. + normal oral microbiota	1
Serratia rubidea	1
Burkholderia gladioli	1
Pseudomonae aeruginosa	1
Candida spp.	2
Candida spp. + normal oral microbiota	1

Table S2. Microorganisms isolated from the tonsillar crypts of 29 patients with a PTA.

**Combinations of Isolated Strains**	**Count (n=29)**
Normal oral microbiota	5
S. aureus	1
S. aureus + normal oral microbiota	1
S. aureus + *Staphylococcus capitis*	1
S. aureus + *Staphilococcus epidermidis*	1
S. aureus + *Candida* spp.	1
S. aureus + *Escherichia coli* + *Candida* spp.	1
S. aureus + *Streptococcus oralis* + *Streptococcus mitis* + *Streptococcus sinensis* + normal oral microbiota	1
Staphilococcus epidermidis	1
Klebsiella pneumoniae	2
Klebsiella pneumoniae + *Candida* spp.	2
Klebsiella oxytoca + *Candida* spp. + normal oral microbiota	1
Acinetobacter ewoffi + normal oral microbiota	1
Bacillus cereus + *Prevotella intermedius*	1
Candida spp.	5
Candida spp. + normal oral microbiota	3
Candida spp. + *Actinomyces*	1
